# Machine Learning-Based Algorithm for Tacrolimus Dose Optimization in Hospitalized Kidney Transplant Patients

**DOI:** 10.3390/diagnostics15232948

**Published:** 2025-11-21

**Authors:** Dong Jin Park, Mihyeong Kim, Hyungjin Cho, Jung Soo Kim, Jeongkye Hwang, Jehoon Lee

**Affiliations:** 1 Department of Laboratory Medicine, Eunpyeong St. Mary’s Hospital, College of Medicine, The Catholic University of Korea, Seoul 03083, Republic of Korea; parkdj1280@gmail.com; 2 Division of Vascular and Transplant Surgery, Department of Surgery, Eunpyeong St. Mary’s Hospital, College of Medicine, The Catholic University of Korea, Seoul 03083, Republic of Korea; mhkim@catholic.ac.kr (M.K.); madeinjoma@naver.com (H.C.); 3 Industry-Academic Cooperation Foundation, The Catholic University of Korea, Seoul 03083, Republic of Korea; wjdtnkim@gmail.com

**Keywords:** tacrolimus, kidney transplantation, machine learning, therapeutic drug monitoring, artificial intelligence

## Abstract

**Background**: Tacrolimus is a cornerstone immunosuppressant in kidney transplantation, but its narrow therapeutic index and marked inter-patient variability complicate dose optimization. Conventional therapeutic drug monitoring (TDM) relies on empirical adjustments that often overlook individual pharmacokinetics. Machine learning (ML) offers a precision dosing alternative by integrating diverse clinical and biochemical variables into predictive models. **Methods**: We retrospectively analyzed 1351 data points from 87 kidney transplant patients at Eunpyeong St. Mary’s Hospital (April 2019–November 2023). Clinical, demographic, and laboratory information, including tacrolimus trough levels and dosing history, were extracted from electronic medical records. Four predictive models—XGBoost, CatBoost, LightGBM, and a multilayer perceptron (MLP)—were trained to forecast next-day tacrolimus concentrations, and model serum creatinine level performance was evaluated using R-squared (R^2^), mean absolute error (MAE), and root-mean-squared error (RMSE). An ensemble model with weighted soft voting was applied to enhance predictive accuracy, and model interpretability was assessed using SHapley Additive exPlanations (SHAP). **Results**: The ensemble model achieved the best overall performance (R^2^ = 0.6297, MAE = 1.0181, RMSE = 1.2999), outperforming all individual models, whereas the MLP model showed superior predictive power among single models, reflecting the significance of nonlinear interactions in tacrolimus pharmacokinetics. SHAP analysis highlighted prior tacrolimus levels, cumulative dose, renal function markers (eGFR level, serum creatinine level), and albumin concentration as the most influential predictors. **Conclusions**: We present a robust ML-based algorithm for tacrolimus dose optimization in hospitalized kidney transplant recipients. By improving predictions of tacrolimus concentrations, the model may help reduce inter-patient dose variability and lower the risk of nephrotoxicity, supporting safer and more individualized immunosuppressive management. This approach advances AI-driven precision medicine in transplant care, offering a pathway to safer and more effective immunosuppression.

## 1. Introduction

Tacrolimus, a cornerstone calcineurin inhibitor in kidney transplantation, is essential for preventing graft rejection. However, its narrow therapeutic index and pronounced inter- and intra-patient variability make dose optimization challenging. Subtherapeutic levels increase rejection risk, while supratherapeutic levels can cause nephrotoxicity and other toxicities [1]. To balance efficacy and safety, therapeutic drug monitoring (TDM) is routinely performed by measuring blood concentrations and adjusting doses. Despite its widespread use, conventional TDM remains largely empirical, often neglecting patient-specific pharmacokinetics and dynamic clinical variables [2].

Machine learning (ML) has emerged as a transformative tool in precision medicine. Applications in imaging, disease classification, and bioinformatics are well established, and more recently, ML has been extended to therapeutic decision making. In drug dosing, ML has shown success in antibiotics such as vancomycin, where models leveraging high-dimensional clinical data improved dose optimization beyond conventional methods [3]. Applying similar strategies to tacrolimus offers potential to enhance patient outcomes by integrating demographic, clinical, and biochemical features into predictive models. Yet, despite these opportunities, few studies have addressed regression-focused ML tasks for tacrolimus dose optimization, leaving a critical gap in the literature [4].

Maintaining therapeutic tacrolimus trough levels is particularly critical during the early post-transplant period, when rejection risk is highest [5]. Current guidelines recommend trough levels of 6–10 ng/mL for optimal outcomes, but retrospective studies suggest that many patients fail to achieve these targets without frequent dose adjustments [6]. This reflects the limitations of empirical dose titration and underscores the need for advanced, data-driven tools to support clinicians in real time.

While artificial intelligence (AI) research in medicine has largely focused on diagnostic imaging and large-scale population datasets, applications in inpatient pharmacological management remain limited [7,8]. Monitoring tacrolimus is especially complex because it requires continuous prediction of drug concentrations rather than categorical classification of outcomes. Such tasks demand models that can capture nonlinear interactions and temporal dynamics in pharmacokinetics. Ensemble approaches—combining algorithms such as XGBoost, LightGBM, and multilayer perceptron (MLP)—are well suited to these challenges, having demonstrated strong predictive performance and robustness in other biomedical domains [9,10]. Importantly, interpretability is crucial for clinical adoption. Tools such as SHapley Additive exPlanations (SHAP) can identify which patient features drive model predictions, providing transparency into how factors such as albumin concentration, serum creatinine level, or prior drug levels influence tacrolimus clearance [11,12].

This study aims to address the unmet need for precision dosing in hospitalized kidney transplant patients by developing and validating an ML-based algorithm for monitoring tacrolimus. Using clinical, demographic, and laboratory data, the algorithm predicts next-day trough concentrations and recommends patient-specific adjustments. By leveraging ensemble learning, the approach seeks to balance predictive accuracy with interpretability, aligning with the goals of precision medicine to improve safety, reduce complications, and optimize therapeutic efficacy [13,14].

Beyond its immediate clinical relevance, this work illustrates the broader role of AI in advancing pharmacological management. Despite major progress in AI for diagnostics, applications that directly impact day-to-day inpatient care—such as real-time dose optimization—remain underexplored [15,16,17]. By focusing on tacrolimus, a widely used immunosuppressant in kidney transplantation that requires careful monitoring, this study highlights how ML can bridge the gap between empirical practices and data-driven decision support.

Looking forward, integrating pharmacogenetic information and validating predictive algorithms across multicenter cohorts will be essential for enhancing model generalizability and clinical utility [18,19,20,21]. Recent studies have explored Bayesian and hybrid PK–ML strategies for tacrolimus dose optimization [6,14,22], highlighting the emerging role of AI in therapeutic drug monitoring. Building on this foundation, our inpatient longitudinal framework addresses real-time, day-to-day dose adjustments within the hospital EHR/LIS environment. Nevertheless, the present study provides a critical first step toward AI-driven precision dosing in transplant care, demonstrating that ML models, coupled with interpretability frameworks, can meaningfully augment traditional TDM practices.

## 2. Method

### 2.1. Study Design and Data Preprocessing

This retrospective observational study was conducted at Eunpyeong St. Mary’s Hospital from April 2019 to November 2023. This study was reviewed and approved by the Institutional Review Board (IRB) of Eunpyeong St. Mary’s Hospital, College of Medicine, The Catholic University of Korea (IRB No. PC23RESI0127) (approval date: 27 July 2023). The requirement for informed consent was waived owing to the retrospective nature of the study, and all procedures were conducted in accordance with the Declaration of Helsinki.

We analyzed data from kidney transplant patients who received tacrolimus as part of immunosuppressive therapy. A total of 87 patients were included in this study, and 1351 data points were used. The data were extracted from the hospital’s electronic medical record (EMR) and laboratory information system (LIS).

The major variables included demographic information (age, gender, weight); clinical parameters (vital signs: blood pressure, heart rate, respiratory rate, and body temperature); and laboratory test results, including tacrolimus trough levels, complete blood count, and various biochemical parameters such as renal function tests (serum creatinine level, eGFR, blood urea nitrogen [BUN]), liver function tests (AST, ALT, total bilirubin, albumin, and total protein levels), and inflammatory markers (C-reactive protein [CRP] and white blood cell count). In total, 71 parameters were used to develop the ML model, with the primary prediction target being the next-day tacrolimus concentration. The route of administration was analyzed only using data on PO medication.

To capture the longitudinal pattern of tacrolimus pharmacokinetics, seven dose-related variables were incorporated into the model. These were “Previous Dose per day,” which represents the total tacrolimus dose administered on the previous day; “Dose_Cumulative sum,” which reflects the cumulative total tacrolimus dose administered up to the day before the prediction; and “FK_506_Cumulative sum,” which indicates the cumulative sum of measured tacrolimus concentration values up to the day before the prediction, in addition to four lagged tacrolimus trough levels: “FK_506_one day before” (tacrolimus trough level one day prior), “FK_506_Two days before” (two days prior), “FK_506_3 days before” (three days prior), and “FK_506_1 week before” (one week prior). These features were designed to enhance the model’s ability to predict next-day tacrolimus concentrations by capturing both cumulative exposure and recent dose–response relationships.

These variables were part of the 71 total parameters used in the model’s development. The model aimed to improve the accuracy of predicting tacrolimus concentrations by integrating these temporal and dosage-related parameters.

The study utilized a total of 95,921 data points, with missing values accounting for 19,104 (19.9%). Three ML models (XGBoost, CatBoost, and LightGBM) and one deep learning model (multilayer perceptron, MLP) were employed. All machine learning models were implemented in Python using XGBoost (v1.6.1), LightGBM (v3.3.2), and CatBoost (v1.0.6). For the three ML models, missing values were left unchanged, allowing the models to estimate values independently; conversely, for the MLP model, missing values were imputed using the median value.

This study aimed to develop an artificial intelligence model to predict the next-day tacrolimus trough level in hospitalized patients using demographic factors (gender, height, weight), clinical parameters (vital signs), and a range of laboratory test results, including cumulative tacrolimus dosing patterns.

Continuous variables are expressed as mean ± standard deviation (SD), while categorical variables are reported as counts with corresponding percentages. Normally distributed continuous variables (e.g., age, height, weight, laboratory parameters) were compared between groups using the independent *t*-test, while categorical variables (e.g., sex) were analyzed using the Chi-square test. The significance level was set at *p* < 0.05 for all statistical tests, and all analyses were conducted using IBM SPSS version 26.0 (SPSS, Chicago, IL, USA).

### 2.2. Machine Learning Models Used, Data Partitioning, and Learning

The initial tacrolimus dose was determined empirically based on the patient’s age, gender, height, and weight. Conversely, dose adjustments were based on the daily monitoring of renal function and tacrolimus concentration through serum creatinine levels. In this study, four ML and deep learning models were used to predict the tacrolimus concentration of the next day using 79 parameters representing the aforementioned factors. These models included three regression models—eXtreme Gradient Boosting (XGBoost), Categorical Boosting (CatBoost), and Light Gradient Boosting Machine (LGBM)—and a deep learning multilayer perceptron (MLP) model. The tacrolimus drug concentration lag variable and cumulative dose indicator were integrated using the feature extraction technique to improve the temporal data representation. The continuous variables were standardized to ensure a consistent scale across features. The stratified K-fold method was used to train the model, with n_split = 10; thus, all the data were used for training using the out-of-fold (OOF) method by dividing the training and validation data at a 9:1 ratio and training for 10 splits. Stratified k-fold cross-validation was used to optimize the model hyperparameters and maximize the prediction performance.

For improved methodological clarity, key hyperparameters and training strategies for each model are summarized below. CatBoostRegressor was trained with a learning rate of 0.05 and 10,000 iterations; XGBRegressor used 1000 estimators with colsample_bytree = 0.9, subsample = 0.8, and colsample_bylevel = 0.8; and LightGBM was trained with 1000 estimators and a colsample_bytree of 0.9. For all three gradient boosting models, early stopping with 50 rounds was applied within each fold during 10-fold cross-validation. The multilayer perceptron (MLP) architecture consisted of two hidden layers (1024 and 512 units) with ELU activation and a 50% dropout rate, followed by a final linear output layer.

### 2.3. Model Performance Evaluation and Ensemble Model Development

Model performance was evaluated using R-squared (R^2^) to measure the explained variance, mean absolute error (MAE) to evaluate the average prediction error, and root-mean-squared error (RMSE) to evaluate the sensitivity to large errors. Model performance was evaluated using R^2^, MAE, and RMSE, averaged over 10-fold cross-validation, with results reported as mean ± standard deviation. These indicators provided a comprehensive evaluation of the predictive ability of the model. XGBoost, CatBoost, LGBM, and MLP were combined using weighted soft voting to enhance the final prediction performance.

### 2.4. SHAP Analysis

SHAP analysis was used to interpret the significance of various parameters used in this study. New variables, including “FK_506_one day before,” “Previous Dose per day,” and Dose_Cumulative_sums, were developed through feature extraction techniques and used to improve the model performance. The SHAP technique provides insight into the contribution of each parameter to the model performance, where SHAP values emphasize the magnitude and directionality of the impact of each feature on the prediction, thereby facilitating transparent and clinically relevant interpretation.

### 2.5. Feature Importance Technique

Feature importance was assessed for the three gradient boosting models—XGBoost, CatBoost, and LightGBM—to evaluate the contribution of individual features to the predictive performance of the models. This analysis focused on identifying the features that influenced model predictions most significantly based on their usage and impact during training.

Feature importance metrics were derived using model-specific methods, such as the number of times a feature was selected for a split or the degree to which it reduced the loss function of the model. Visualizations of feature importance were generated for each model to provide an overview of the relative significance of the predictors. This approach enabled a systematic comparison of feature relevance across the three ML models.

This study ensured a robust and data-driven foundation for model evaluation and subsequent interpretation by integrating the insights gained from feature importance analysis with the overall modeling framework. The analysis complemented the SHAP-based interpretability techniques used in the study, providing a dual perspective on the factors influencing model performance.

## 3. Results

### 3.1. Patient Demographic Characteristics and Tacrolimus Concentration

The data for this study included demographic parameters (height, weight, gender, age); vital signs; and various laboratory test results (Supplementary Table S1).

**Figure 1:**
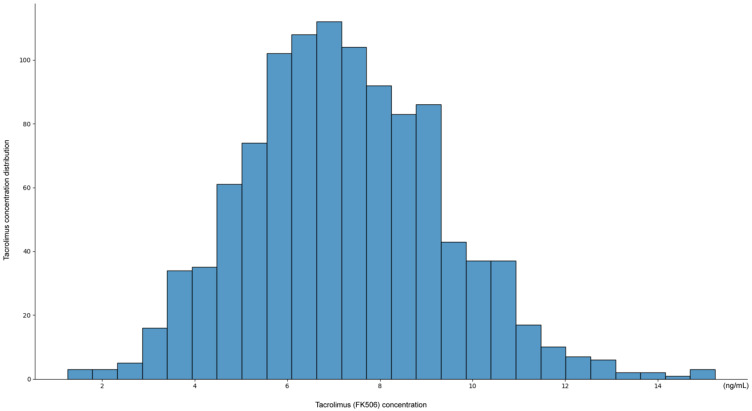
Tacrolimus (FK506) dose optimization in hospitalized kidney transplant patients (ng/mL).

The distribution of tacrolimus concentrations exhibited a unimodal pattern, with most measurements concentrated within the therapeutic window of 6–10 ng/mL (Figure 1). The peak frequency was observed around 7–8 ng/mL, suggesting that current dosing strategies generally maintain tacrolimus levels within this range.

However, the distribution showed a slight skew, with a subset of patients exhibiting subtherapeutic levels (<6 ng/mL), with some values as low as 2 ng/mL, and a smaller subset of patients exhibiting supratherapeutic levels (>12 ng/mL), with occasionally values reaching 14 ng/mL. These findings indicate that while most patients maintain therapeutic tacrolimus levels, individual variability remains, emphasizing the need for personalized dosing adjustments.

Analysis of demographic characteristics revealed significant differences between deceased donor and living donor kidney transplant recipients. Age distribution showed a statistically significant difference, with the deceased donor group being older (62.12 ± 7.40 years) compared with the living donor group (53.00 ± 10.49 years; *p* < 0.001).

By contrast, no significant differences were observed in gender distribution (*p* = 1.000), suggesting that sex may not be a major determinant in donor type classification. Similarly, body weight (67.04 ± 11.18 vs. 69.98 ± 14.43 kg; *p* = 0.309) and height (162.96 ± 15.89 vs. 166.48 ± 8.62 cm; *p* = 0.188) did not differ significantly between deceased and living donor recipients.

These findings suggest that age is a key differentiating factor between deceased and living donor kidney transplant recipients, while other demographic variables such as sex, height, and weight appear to be comparable across both groups.

The observed distribution pattern of tacrolimus concentrations highlights the challenge of maintaining consistent therapeutic levels and underscores the potential value of an AI-based approach for personalized dosing recommendations.

### 3.2. Performance of Artificial Intelligence Models

Table 1 presents the performance of various ML models in predicting tacrolimus concentrations for kidney transplant patients, including XGBoost, CatBoost, LGBM, MLP (deep learning), and ensemble models that utilize weighted soft voting methods.

**Figure 2:**
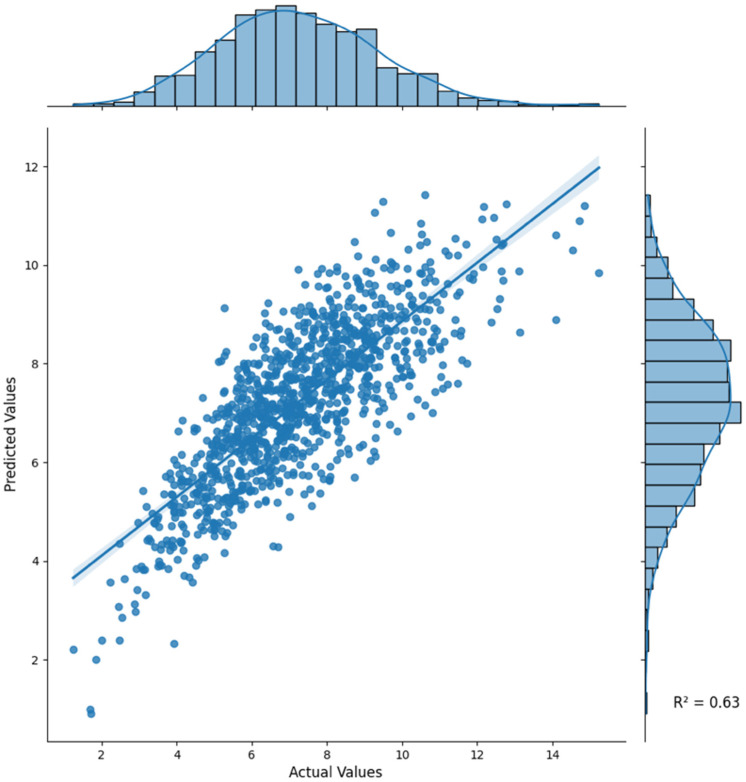
Ensemble model. The ensemble model combining XGBoost, CatBoost, LightGBM, and MLP demonstrated superior performance, achieving the highest R^2^ (0.6297), highlighting the value of ensemble learning in improving prediction accuracy.

The ensemble model showed the highest performance in all evaluation metrics, achieving an R^2^ of 0.6297, MAE of 1.0181, and RMSE of 1.2999 (Table 1, Figure 2); therefore, this model is more accurate and reliable compared with individual models.

Model evaluation was conducted using an out-of-fold 10-fold cross-validation framework for internal validation. Performance variability across folds was minimal (Table 1), confirming the robustness of the ensemble model. As this study was conducted using single-center data without an external validation cohort, model generalizability should be interpreted within this context.

As summarized in Table 1, the ensemble model achieved the highest overall performance with the lowest error and narrowest variability across 10-fold cross-validation, indicating stable and reproducible predictions. Among the individual models, the deep learning MLP model performed the best, with an R^2^ of 0.6016, MAE of 1.0484, and RMSE of 1.3484.

Among the three ML models, CatBoost performed the best with an R^2^ of 0.5802, MAE of 1.0936, and RMSE of 1.3842, followed by XGBoost and LGBM in that order.

The deep learning MLP model outperformed the three ML models, effectively capturing linear and nonlinear relationships in data, including among various blood test results and tacrolimus concentration and dose, thus overcoming the limitations of the tree-based ML models in addressing complex interactions in structured medical datasets. Additionally, the deep learning MLP model was confirmed to be usable on hospital structured data as a boosting-based tree-based regression model. Outliers were removed to generalize and optimize model performance. In the ensemble technique, most weight was placed on deep learning using the soft-voting technique for the prediction results to optimize the model, followed by the CatBoost model. The lowest weight was assigned equally to XGBoost and LGBM.

### 3.3. Comprehensive SHAP Analysis

SHAP analysis facilitates an understanding of the contribution of features to the four ML models. The results of this analysis highlight the importance of recent tacrolimus dose, cumulative clinical observations, and patient-specific biochemical and physiological characteristics. By integrating the aforementioned variables, the models have the potential to improve personalized dosing strategies.

#### 3.3.1. XGBoost (SHAP Analysis)

**Figure 3:**
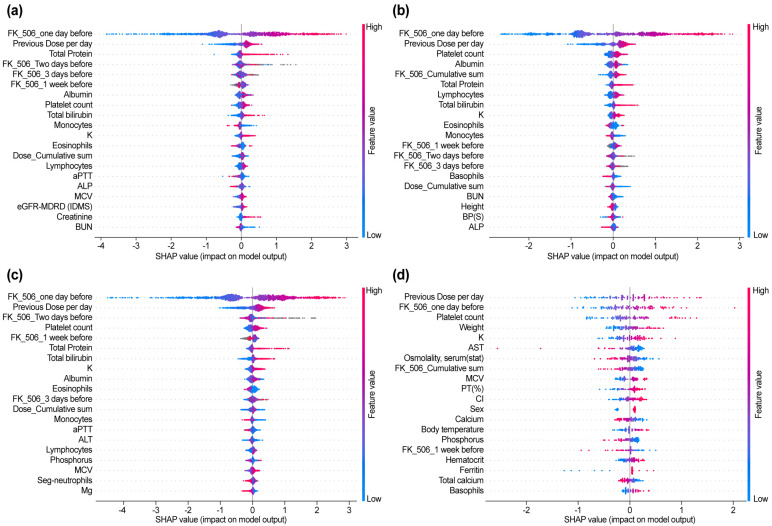
SHAP summary plots for each model. SHAP (SHapley Additive exPlanations) values for key features predicting hospitalized kidney transplant patients across four models: (**a**) XGBoost, (**b**) CatBoost, (**c**) LGBM, and (**d**) MLP. The plots show the impact of “FK_506_1 day ago” as the most influential predictors. Abbreviations: FK_506 = tacrolimus; AST = aspartate aminotransferase; ALT = alanine aminotransferase; MCV = mean corpuscular volume; PT = prothrombin time; eGFR = estimated glomerular filtration rate.

XGBoost (Figure 3a) identified “FK_506_one day before” as the most important feature, indicating a strong positive correlation with the predicted tacrolimus concentration. The SHAP values highlighted that a higher tacrolimus concentration the day before (shown in red) significantly contributes to a higher tacrolimus concentration the next day, which is the final predicted value, while a lower concentration (shown in blue) results in a lower predicted value. The second most influential feature, “Previous dose per day,” emphasized that the dose plays a direct role in predicting future tacrolimus concentrations. Temporal variables, including “FK_506_Two days before” and “FK_506_3 days before,” were also found to be significant, reflecting the importance of sequential dose data in understanding pharmacokinetics. Biochemical parameters, such as total protein level, albumin concentration, and platelet count, provide additional physiological context by capturing the variability in drug absorption, metabolism, and elimination. The ability of XGBoost to integrate both temporal and physiological features is particularly effective for modeling tacrolimus pharmacokinetics.

#### 3.3.2. CatBoost (SHAP Analysis)

CatBoost (Figure 3b), similar to XGBoost, identified “FK_506_one day before” and “Previous Dose per day” as the most crucial predictors. The unique feature of this model is that—similar to the other two ML models (XGBoost, LGBM)—it focuses on “Dose_Cumulative_sum”; however, in terms of SHAP color, it showed a pattern in which the cumulative dose increased and the predicted drug concentration decreased. This can be attributed to individual patient differences and reflects the clinical adjustment of the dose during long-term hospitalization to maintain the therapeutic range.

Other important variables include platelet count, albumin concentration, and total protein level, which reaffirm the relevance of biochemical indicators. CatBoost’s emphasis on the pattern of cumulative dose highlights its usefulness in tracking long-term medication management strategies and their clinical implications.

#### 3.3.3. LGBM (SHAP Analysis)

LGBM (Figure 3c), similar to other tree-based models, identified “FK_506_one day before” and “Previous Dose per day” as the largest contributors. This model, like other ML models, prioritizes immune system markers such as lymphocytes and monocytes, suggesting their role in tacrolimus metabolism and elimination. Liver function markers, including total potassium level and renal function indicators, such as K (potassium), also emerged as key features, highlighting the interaction between organ function and drug pharmacokinetics. Temporal features, such as “FK_506_2 days ago” and “FK_506_3 days ago,” highlighted the strength of the model in utilizing historical data for prediction. SHAP values revealed that high bilirubin levels (red), indicative of liver dysfunction, contributed to increased predicted tacrolimus concentrations, while electrolytes such as potassium level provided insights into electrolyte balance during treatment.

#### 3.3.4. MLP (Deep Learning) (SHAP Analysis)

The MLP model (Figure 3d) demonstrated its ability to capture complex, nonlinear interactions, yielding a broader distribution of feature importance compared with that of tree-based models. Key predictors included “Previous Dose per day” and “FK_506_one day before,” reinforcing the significance of prior tacrolimus exposure in determining next-day concentrations.

Distinct from tree-based models, MLP incorporated additional metabolic and hematologic markers, such as “Platelet count,” “Weight,” “Potassium (K),” and “AST,” which reflect broader physiological processes relevant to tacrolimus pharmacokinetics. Additionally, “Osmolality, serum (stat),” “FK_506_Cumulative sum,” and “MCV” were recognized as influential variables, emphasizing the importance of cumulative exposure and blood composition in drug metabolism.

Renal and electrolyte markers, including “magnesium,” “Calcium,” and “Body temperature,” also played significant roles, highlighting the MLP model’s capacity to integrate diverse physiological factors beyond direct renal function indicators. Unlike tree-based models, which primarily focused on sequential tacrolimus dosing patterns, the MLP model effectively captured subtle relationships between metabolic parameters and immunosuppressant levels.

These findings suggest that MLP is particularly well suited for tacrolimus dose optimization in hospitalized kidney transplant patients, as it accounts for both traditional pharmacokinetic parameters and broader metabolic interactions. By leveraging these insights, MLP enables a more individualized approach to tacrolimus dosing, potentially reducing variability and improving patient outcomes.

### 3.4. SHAP Color Interpretation and Clinical Relevance

In all models, SHAP values (high feature values are shown in red, low feature values in blue) provided intuitive insights into the directional influence of each feature. For instance, in all models, higher pre-dose trough concentrations (red) led to higher predicted concentrations, highlighting the importance of immediate pre-dose data. Similarly, features such as “Dose_Cumulative_sum” in CatBoost are considered to be caused by individual differences in patients. Different concentrations of tacrolimus can lead to varying cumulative doses in patients, highlighting the necessity for additional genetic evaluation of individual patients. The SHAP analysis was performed for all four AI models, and by visualizing the contribution of features as such, the SHAP analysis provided actionable clinical insights into the factors driving tacrolimus pharmacokinetics.

All models consistently identified “FK_506_one day before” and “Previous Dose per day” as the most influential predictors, reinforcing the significance of prior tacrolimus exposure in determining next-day concentrations. The three tree-based models (XGBoost, CatBoost, and LGBM) placed considerable emphasis on sequential dose-related variables, including “FK_506_Two days before,” “FK_506_3 days before,” and “FK_506_1 week before,” alongside “Dose_Cumulative sum,” which reflects cumulative dose history.

Conversely, the MLP model prioritized “FK_506_Cumulative sum” rather than “Dose_Cumulative sum.” Additionally, MLP uniquely incorporated “Body temperature,” “Hematocrit,” “Ferritin,” “Total calcium,” “Chloride (Cl),” “Weight,” and “PT (%),” which were not prominently selected in the tree-based models. This result suggests that while tree-based models primarily rely on dose–response relationships, MLP captures more complex physiological interactions, integrating metabolic and systemic markers.

These findings indicate that tree-based models excel in leveraging structured dose–response patterns, while MLP enhances predictive power by incorporating broader physiological features. Combining both approaches may offer complementary insights for optimizing tacrolimus dosing in kidney transplant patients.

### 3.5. Feature Importance Results by Model

#### 3.5.1. XGBoost (Feature Importance)

**Figure 4:**
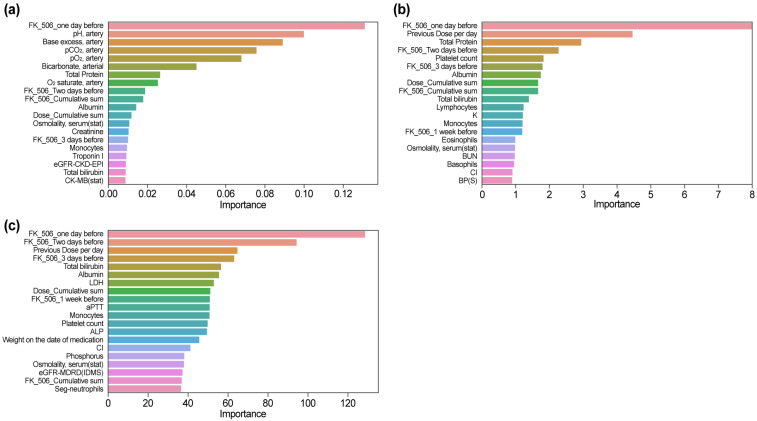
Feature importance results by model. Relative importance of predictive features for tacrolimus concentration across three models: (**a**) XGBoost, (**b**) CatBoost, (**c**) and LGBM. Abbreviations: BP(S), systolic blood pressure; BUN, blood urea nitrogen; eGFR, estimated glomerular filtration rate; MDRD, Modification of Diet in Renal Disease; CKD-EPI, Chronic Kidney Disease Epidemiology Collaboration; FK_506, tacrolimus.

In the XGBoost model (Figure 4a), the feature importance ranking revealed a unique emphasis on parameters related to arterial blood gas analysis. “pH, artery” and “Base excess, artery” were ranked second and third, respectively, after “FK_506_one day before.” Other arterial parameters, such as “pCO_2_, artery,” “pO_2_, artery,” and “Bicarbonate, arterial,” were also highly ranked, indicating the sensitivity of the model to acid–base balance and respiratory markers. Biochemical markers like “Total Protein,” “Albumin,” and “Creatinine” were ranked among the top predictors, highlighting their relevance to tacrolimus pharmacokinetics. Notably, while “FK_506_one day before” remained influential, its relative importance was lower compared with SHAP analysis. Temporal features, such as “FK_506_Two days before” and “FK_506_3 days before,” and cumulative metrics such as “Dose_Cumulative sum” were also included, reflecting their utility in integrating longitudinal dosing data.

#### 3.5.2. CatBoost (Feature Importance)

In the CatBoost model (Figure 4b), “FK_506_one day before” and “Previous Dose per day” were consistently identified as the most critical features, highlighting their immediate relevance to predicting next-day tacrolimus concentrations. Biochemical markers such as “Total Protein” and “Albumin” were also ranked highly, indicating their role in drug absorption and metabolism. Temporal variables, including “FK_506_Two days before” and “FK_506_3 days before,” underscored the importance of sequential dosing patterns. Additional features such as “Platelet count” and “Lymphocytes” were uniquely emphasized, highlighting the capacity of the model to incorporate immune-related indicators into its predictions. The cumulative measure “Dose_Cumulative sum” was moderately ranked, suggesting its role in providing a broader context for dosing trends. Overall, CatBoost demonstrated a balanced integration of immediate, temporal, and physiological parameters, thus enhancing its capability for modeling tacrolimus pharmacokinetics.

#### 3.5.3. LightGBM (Feature Importance)

The LightGBM model (Figure 4c) highlighted “FK_506_one day before,” “Previous Dose per day,” and “FK_506_Two days before” as its most significant features, reflecting the reliance of the model on immediate and sequential dosing data for tacrolimus concentration predictions. In addition to these temporal variables, the model also ranked “Total Bilirubin,” “Albumin,” and “LDH” prominently, emphasizing the role of biochemical markers in drug metabolism and clearance. A notable distinction in LightGBM was the higher ranking of “Weight on the date of medication,” which differentiated it from other models by incorporating patient-specific physical characteristics into the prediction process. Immune markers such as “Monocytes” and “Platelet count,” along with cumulative measures like “Dose_Cumulative sum” and renal function indicators like “eGFR-MDRD (IDMS),” were moderately ranked. LightGBM’s ability to integrate diverse clinical and physiological data, including unique variables like patient weight, demonstrated its robustness and flexibility in tacrolimus pharmacokinetics modeling.

### 3.6. Significant Clinical and Biochemical Differences Between Therapeutic and Non-Therapeutic States

Significant differences were observed between therapeutic and non-therapeutic tacrolimus concentration states across multiple biochemical parameters based on 1351 data points collected from 87 kidney transplant patients (Supplementary Table S2). As each patient had multiple measurements over their hospitalization period, these comparisons reflect dynamic changes in laboratory values corresponding to different tacrolimus concentration ranges.

Renal function markers showed notable differences between the two states. Blood urea nitrogen (BUN) (27.88 ± 16.89 vs. 35.48 ± 20.15 mg/dL, *p* < 0.0001) and serum creatinine level (1.96 ± 1.98 vs. 3.12 ± 2.75 mg/dL, *p* < 0.0001) were significantly lower when tacrolimus concentrations were within the therapeutic range, while eGFR values (eGFR-MDRD: 55.91 ± 30.89 vs. 39.72 ± 30.66 mL/min/1.73 m^2^, *p* < 0.0001; eGFR-CKD-EPI: 58.33 ± 30.49 vs. 43.22 ± 32.79 mL/min/1.73 m^2^, *p* < 0.0001) were higher, indicating better renal function.

Electrolyte and metabolic markers also showed significant differences. Serum phosphorus levels were lower during therapeutic tacrolimus states (2.72 ± 1.52 vs. 3.41 ± 1.89 mg/dL, *p* < 0.0001), whereas potassium level (4.40 ± 0.67 vs. 4.20 ± 0.64 mmol/L, *p* < 0.0001) and chloride level (105.71 ± 4.85 vs. 104.41 ± 5.58 mmol/L, *p* < 0.0001) were slightly elevated. Serum magnesium was also lower when tacrolimus concentrations were within the therapeutic range (1.83 ± 0.31 vs. 1.94 ± 0.35 mg/dL, *p* < 0.0001).

Hematological parameters reflected immune-related differences, with higher lymphocyte percentages (8.46 ± 5.54% vs. 7.29 ± 5.46%, *p* < 0.0001) and monocyte percentages (5.73 ± 2.83% vs. 5.18 ± 2.79%, *p* < 0.001) observed when tacrolimus levels were within the therapeutic range.

Lastly, serum osmolality was lower during therapeutic tacrolimus states (294.03 ± 9.42 vs. 296.84 ± 10.21 mOsm/kg, *p* < 0.0001), suggesting potential differences in hydration status or electrolyte balance depending on tacrolimus levels.

These findings indicate that maintaining tacrolimus concentrations within the therapeutic range is associated with better renal function, stable electrolyte balance, and improved immune regulation. The observed variations across different tacrolimus concentration states highlight the importance of individualized dosing strategies to optimize patient outcomes.

## 4. Discussion

This study demonstrates that ML can substantially improve tacrolimus monitoring by leveraging multidimensional patient data to enable individualized, real-time dose optimization. Using demographic, clinical, and biochemical variables, we developed predictive models that accurately estimated next-day tacrolimus trough concentrations, addressing a longstanding challenge in the management of this essential immunosuppressant.

The ensemble model combining XGBoost, CatBoost, LightGBM, and multilayer perceptron (MLP) achieved the highest predictive performance (R^2^ = 0.6297, MAE = 1.0181), underscoring the benefit of integrating complementary algorithms [10,22,23]. Ensemble learning, by aggregating predictions from diverse models, mitigates individual model limitations and improves robustness—an approach consistent with prior reports showing that ensembles outperform single models in complex regression tasks [9,24].

Clinical observations reinforced the importance of individualized dosing. Patients with greater fluctuations in tacrolimus concentrations also showed parallel variability in renal function indicators such as serum creatinine and eGFR levels, highlighting the risks of unstable drug exposure. In addition, significant changes in laboratory values—including phosphorus levels (2.72 ± 1.52 vs. 3.41 ± 1.89 mg/dL, *p* < 0.001) and osmolality (294.03 ± 9.42 vs. 296.84 ± 10.21 mOsm/kg, *p* < 0.001)—were observed alongside tacrolimus variability. These findings emphasize the need to incorporate dynamic laboratory parameters into predictive models to better capture intra-patient variability. Interestingly, serum osmolality was lower in the therapeutic tacrolimus group. This finding may reflect subtle alterations in tubular function, as tacrolimus is known to induce vasoconstriction and tubular dysfunction that can influence water and electrolyte balance. Such changes could contribute to reduced serum osmolality despite stable drug levels.

Compared with conventional pharmacokinetic approaches, which typically rely on a small set of predictors (age, sex, weight, creatinine, and tacrolimus dose), our model leveraged 76 parameters, substantially improving precision and reliability. The weighted soft-voting ensemble strategy further enhanced performance by integrating the unique strengths of diverse algorithms.

Among the individual models, the MLP consistently outperformed tree-based methods. While gradient-boosting algorithms such as CatBoost and XGBoost are well suited for nonlinear interactions, the MLP’s capacity to process complex physiological and temporal data proved advantageous. Its superior performance likely reflects an ability to capture intricate relationships among immune markers, renal function indicators, and electrolyte balance. This was particularly evident in differentiating between therapeutic and non-therapeutic groups, where significant variations were observed in parameters such as lymphocytes, monocytes, and osmolality.

Overall, these findings highlight the advantages of integrating broad, dynamic clinical datasets with advanced ML techniques for precision dosing. By capturing both cumulative and temporal effects, ML-based models can more accurately reflect the multifactorial determinants of tacrolimus pharmacokinetics and provide a foundation for real-time, individualized dose adjustment.

**Figure 5:**
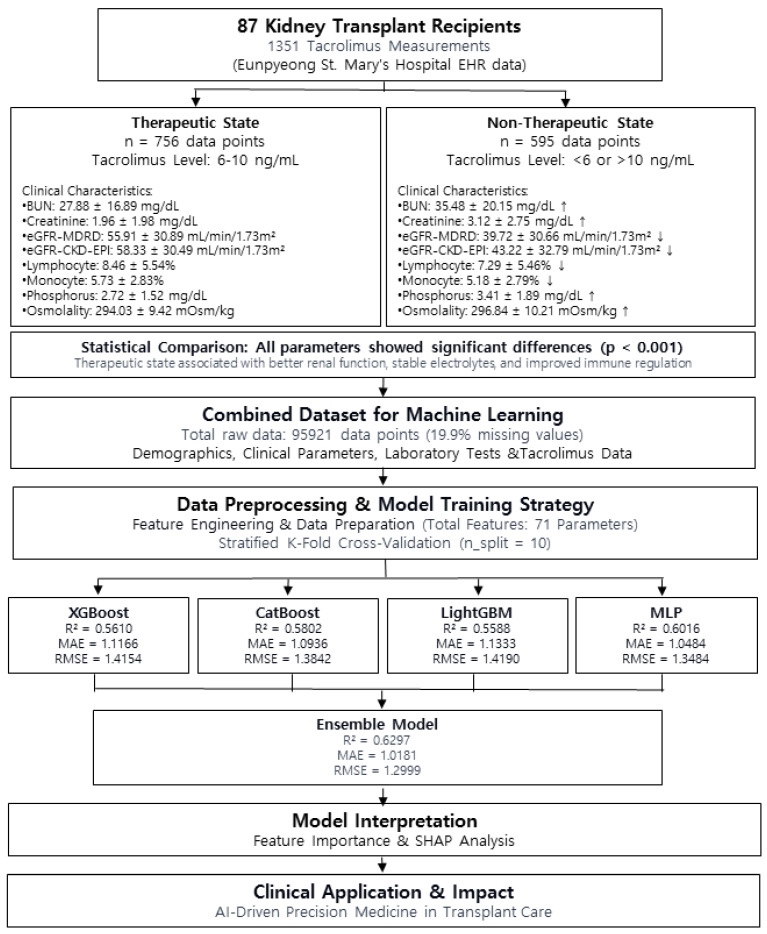
Study flow chart.

To contextualize how these predictive capabilities could be operationalized in clinical practice, Figure 5 outlines a conceptual workflow for integrating the model into hospital EHR/LIS systems for real-time tacrolimus monitoring and dose adjustment.

In contrast to gradient-boosting models, which rely on hierarchical decision trees, MLP captures intricate dependencies across input features, rendering it well suited for complex clinical datasets. MLP uniquely incorporates renal function indicators, including eGFR-MDRD and eGFR-CKD-EPI, further improving its ability to predict individual drug response.

Ensemble modeling combines the complementary strengths of these algorithms to further improve predictive performance [9,24]. This approach is consistent with previous findings showing that ensemble methods can outperform single models in regression tasks requiring high-dimensional feature integration [10].

SHAP analysis and feature importance ranking provided crucial insights into the underlying mechanisms driving model predictions. In all models, the most influential predictors were consistently identified as the most recent tacrolimus dose (“FK_506_1 day ago”) and cumulative dose, highlighting the importance of temporal patterns in pharmacokinetics.

As illustrated in Figure 3 and Figure 4, “FK_506_one day before” and “Previous Dose per day” reflect the time-dependent accumulation pattern of tacrolimus, while lower albumin and higher bilirubin levels were linked to increased concentrations, consistent with reduced protein binding and hepatic metabolic inhibition.

Although tacrolimus is not primarily albumin-bound and instead associates mainly with erythrocytes and α-1 acid glycoprotein, albumin appeared as an influential SHAP predictor because it reflects broader physiological and metabolic status affecting tacrolimus clearance.

Tree-based models emphasized renal and hepatic predictors, whereas the MLP identified additional nonlinear effects related to albumin and cumulative dose, underscoring complementary feature recognition across algorithms.

Clinically, these SHAP-identified features provide practical cues for patient management: recent tacrolimus levels and cumulative exposure reinforce the necessity of dose-history review, renal function markers indicate when closer monitoring or dose reduction may be warranted, and albumin levels reflect overall physiologic reserve that may indirectly influence clearance. These associations offer actionable insights for individualized dosing, while complementing clinical judgment.

SHAP results reflect model-derived feature influence rather than pharmacologic mechanisms, and were interpreted in this study as associations within the predictive framework rather than causal determinants.

Biochemical markers, including albumin concentration, serum creatinine level, and eGFR level, also played an important role, reflecting their relevance to drug absorption, metabolism, and elimination.

Notably, XGBoost highlighted the importance of parameters such as those used in arterial blood gas analysis (e.g., “Base excess, arterial” and “pH, arterial”) in the feature importance analysis, suggesting the potential impact of acid–base balance on tacrolimus pharmacokinetics. Similarly, SHAP analysis showed that cumulative dosing patterns and renal function indicators significantly impacted prediction, reinforcing the clinical understanding that tacrolimus clearance is heavily influenced by kidney function and long-term drug exposure [25,26]. The differences between models provide complementary perspectives on pharmacokinetic variability.

To enhance interpretability, we further clarified the distinction between temporal and physiological features identified by SHAP analysis. Temporal variables such as recent tacrolimus doses and cumulative exposure reflect immediate pharmacokinetic patterns, whereas physiological variables including renal function and albumin levels indicate the patient’s metabolic capacity. Clinically, this balance provides actionable insight—dose reduction or intensified monitoring may be warranted in patients with declining renal function or hypoalbuminemia. Such SHAP-based interpretation bridges model prediction and bedside decision making, facilitating personalized and safer tacrolimus management.

As reflected in the revised Figure 3, Figure 4 and Figure 5, the ensemble model demonstrated stable and clinically interpretable predictions (Table 1), with higher renal and electrolyte abnormalities observed in non-therapeutic states (Figure 5) and clearer visualization of influential predictors such as *FK_506 one day before*, creatinine, and albumin (Figure 3 and Figure 4). These results support the model’s real-world applicability for tacrolimus monitoring and individualized dosing.

As summarized in Supplementary Table S2 and Figure 5, patients in the non-therapeutic group showed higher BUN, creatinine, and phosphorus levels and lower eGFR values compared with those in the therapeutic group, consistent with reduced renal clearance and altered tacrolimus pharmacokinetics. The inclusion of 95% confidence intervals for inter-group differences supports the clinical reliability of these findings and further validates the physiological relevance of the model’s predictions.

As shown in Supplementary Table S2 and Figure 5, patients in the non-therapeutic state exhibited higher BUN, creatinine, and phosphorus levels, and lower eGFR values compared with those in the therapeutic range. These trends support the clinical validity of our model’s key predictors, indicating that impaired renal function and electrolyte imbalance contribute to tacrolimus overexposure or underexposure.

As shown in Table 1 and Figure 5, the ensemble model achieved the highest overall performance with the lowest error and narrowest variability across 10-fold cross-validation, demonstrating stable and reproducible predictions compared with individual algorithms (Supplementary Table S2).

Although this study was conducted in a relatively small kidney transplant cohort, each patient contributed multiple longitudinal measurements, resulting in 1351 tacrolimus trough observations. The large number of data points per outcome, the highly significant between-state differences with narrow confidence intervals for key laboratory markers (Supplementary Table S2), and the small variability of cross-validated performance metrics (Table 1) together suggest that the available sample size was adequate for training and internal validation of the prediction models, while external validation in larger multicenter cohorts remains an important future step.

To provide context, we discuss our model’s performance in relation to results reported from population pharmacokinetic (PK) and Bayesian approaches, including studies by Lloberas et al. [6] and Wang et al. [14], which represent clinical standards for tacrolimus dose prediction. While those models achieved moderate accuracy in structured cohorts, our ensemble ML framework demonstrated comparable or improved performance using real-world data. Rather than replacing PK-based modeling, the ML approach complements it by capturing nonlinear patterns across high-dimensional clinical variables. Integration of PK and ML paradigms may further advance individualized tacrolimus dosing.

In line with recent studies exploring Bayesian and hybrid PK–ML approaches for tacrolimus dosing (Wang et al., 2024 [14]; Basuli and Roy, 2023 [20]), our framework extends these concepts to a real-world hospital setting. As illustrated in the updated Figure 5, this study presents a conceptual workflow for integrating AI-assisted therapeutic drug monitoring into EHR/LISs, supporting individualized dose adjustment in routine transplant care.

The proposed conceptual framework, illustrated in the newly added Figure 5 (study flow chart), outlines how the model could potentially be integrated into hospital EHR/LISs for real-time clinical use. Tacrolimus concentrations, dosing records, and laboratory results could be automatically extracted, analyzed by the model, and presented on a clinician dashboard. When predicted concentrations exceed or fall below the therapeutic range, the system could generate alerts for physician review. Although this integration has not yet been implemented, the framework demonstrates the model’s potential as an explainable decision-support tool to enhance individualized tacrolimus dosing while maintaining clinician oversight.

Another consideration is the potential influence of unmeasured pharmacogenetic and pharmacological factors. Tacrolimus clearance is affected by CYP3A5 polymorphisms, concomitant medications that alter CYP3A activity, and post-transplant phase-related changes in graft function. These factors were not directly included, due to the retrospective nature of the dataset, but may be indirectly reflected by correlated markers such as creatinine, eGFR, and albumin. Future studies incorporating pharmacogenomic and co-medication data will help improve mechanistic interpretability and model precision.

The differences in the observed renal function indicators, including lower serum creatinine levels and higher eGFR level values in the treatment group, are consistent with the known nephrotoxic effects of tacrolimus, which can occur even at therapeutic concentrations due to its impact on pre-glomerular arterioles [27]. Similarly, improvements in hematological parameters, such as hemoglobin level and platelet count, further support the clinical relevance of these predictors reflecting tacrolimus-related outcomes [28].

The integration of time series data, including lagged variables and cumulative tacrolimus dose indicators, represents one of the key contributions of this study. Historical tacrolimus concentrations and accumulated drug exposure played a crucial role in predicting next-day levels, aligning with established pharmacokinetic principles.

Temporal dosing variables, particularly “FK_506_one day before” and “Dose_Cumulative_sum,” were crucial for modeling the sequential drug effect that addresses the dynamic nature of tacrolimus pharmacokinetics. The inclusion of these variables is consistent with recommendations from previous studies that emphasize the importance of temporal data in pharmacokinetic modeling [29,30].

Patient-to-patient variability in tacrolimus metabolism—driven by factors such as renal function, hydration status, and immune markers (e.g., lymphocytes and monocytes)—was effectively captured by ML models. These results highlight the utility of ML in incorporating a variety of clinical and demographic features to account for variability in drug response [31,32]. In addition, reliance on SHAP values to quantify feature contributions increases model transparency, which is an important factor in clinical decision making [33,34].

## 5. Conclusions

This study demonstrates the feasibility of an ML-based framework for optimizing tacrolimus dosing in kidney transplant recipients. By integrating cumulative dosing histories, temporal patterns, and clinical parameters, the model can predict tacrolimus concentrations with high accuracy, offering a data-driven approach to minimize adverse effects and improve therapeutic efficacy. Integration of such models into EHR systems could enable real-time dose recommendations, improve dosing accuracy, and potentially enhance graft and patient survival (Figure 5).

Despite promising results, several limitations must be acknowledged. The single-center, retrospective design may restrict generalizability; multicenter validation is essential to confirm broader applicability [35]. In addition, the single-institution setting may reflect specific clinical workflows that could influence model performance. Tacrolimus therapeutic drug monitoring (TDM) intervals, assay calibration procedures, and post-transplant management protocols often vary between centers, potentially affecting measured trough levels and pharmacokinetic patterns. To mitigate this limitation, we incorporated real-world longitudinal data covering both living- and deceased-donor transplant cases and applied an ensemble framework to enhance adaptability across heterogeneous clinical conditions. Nevertheless, external validation using independent cohorts remains essential, and future work will prioritize multicenter validation to confirm generalizability. Furthermore, pharmacogenetic factors such as CYP3A5 polymorphisms, which substantially influence tacrolimus metabolism, were not included [36,37]. Incorporating pharmacogenomic markers and applying dimensionality reduction strategies could further improve model performance and personalization.

Future work should also evaluate the real-world impact of ML-guided dosing on clinical outcomes, including rejection rates, nephrotoxicity, and hospital length of stay. Prospective studies, coupled with user-friendly EHR interfaces, will be critical for clinical translation and scalability [38,39].

The principles established here extend beyond transplantation. ML-driven TDM has potential applications for other therapeutics requiring precise dosing, such as antibiotics, antifungals, and anticoagulants [40]. Expanding these approaches to incorporate pharmacogenomic and metabolomic data could advance predictive precision and support the broader vision of individualized therapy [41].

In summary, this study provides a robust foundation for AI-assisted precision dosing in transplant medicine. By addressing variability in tacrolimus pharmacokinetics and enabling personalized, data-driven decision making, ML-based TDM represents a pivotal step toward safer, more effective, and scalable therapeutic management.

## Figures and Tables

**Table 1:** Prediction performance for tacrolimus concentration models.

Model	R^2^ (±SD)	MAE (±SD)	RMSE (±SD)
XGBoost	0.5610 ± 0.07	1.1166 ± 0.06	1.4154 ± 0.08
CatBoost	0.5802 ± 0.07	1.0936 ± 0.08	1.3842 ± 0.09
LGBM	0.5588 ± 0.06	1.1333 ± 0.07	1.4190 ± 0.08
MLP (Deep Learning)	0.6016 ± 0.10	1.0484 ± 0.09	1.3484 ± 0.10
Ensemble (Weighted Soft Voting)	0.6297 ± 0.08	1.0181 ± 0.07	1.2999 ± 0.08

R^2^: R-squared ± standard deviation (SD); MAE: mean absolute error; RMSE: root-mean-squared error; XGBoost: eXtreme Gradient Boosting; LGBM: Light Gradient Boosting Machine; CatBoost: Category Boosting; MLP: multilayer perceptron (deep learning); ensemble (weighted soft voting).

## Data Availability

The original contributions presented in this study are included in the article/Supplementary Material. Further inquiries can be directed to the corresponding authors.

## Supplementary Materials

The following supporting information can be downloaded at https://www.mdpi.com/article/10.3390/diagnostics15232948/s1, Table S1: Comparison of clinical characteristics between deceased donor and living donor kidney transplant recipients; Table S2: Significant laboratory parameters associated with non-therapeutic and therapeutic tacrolimus concentration states based on 1351 data points from 87 kidney transplant patients.
