# Supplementary material for: Machine Learning-Based Algorithm for Tacrolimus Dose Optimization in Hospitalized Kidney Transplant Patients
Source: Diagnostics (Basel). 2025 Nov 21;15(23):2948. doi: 10.3390/diagnostics15232948 (PMC12691189; doi:10.3390/diagnostics15232948)
Supplement: Supplementary file 1 [file diagnostics-15-02948-s001.zip › HQJMH_4_4_Supplemen_Table S1_20250929.pdf]

Supplementary Table S1. Comparison of clinical characteristics between deceased donor and living donor kidney transplant recipients

Variables	Deceased donor Mean ± SD (n = 51)	Living donor Mean ± SD (n = 36)	*P*-value
**Sex**			
Female	41.2 (%) (n=21)	41.7 (%) (n=15)	1.000
Male	58.8 (%) (n=30)	58.3 (%) (n=21)	
Height (cm)	162.96 ± 15.89	166.48 ± 8.62	0.188
Weight (kg)	67.04 ± 11.18	69.98 ± 14.43	0.309
Age (years)	62.12 ± 7.40	53.00 ± 10.49	< 0.001

Continuous variables are expressed as mean ± standard deviation (SD), and categorical variables as counts (percentages). Normally distributed continuous variables (e.g., age) were analyzed using an independent t-test. Categorical variables (e.g., sex) were compared using the Chi-square test.
