# Supplementary material for: Machine Learning-Based Algorithm for Tacrolimus Dose Optimization in Hospitalized Kidney Transplant Patients
Source: Diagnostics (Basel). 2025 Nov 21;15(23):2948. doi: 10.3390/diagnostics15232948 (PMC12691189; doi:10.3390/diagnostics15232948)
Supplement: Supplementary file 1 [file diagnostics-15-02948-s001.zip › Supplement_Table S2_20251105_UPLOAD.pdf]

Supplementary Table S2. Significant laboratory parameters associated with non-therapeutic and therapeutic tacrolimus concentration states based on 1,351 data points from 87 kidney transplant patients

Significance Parameters (Total Parameters No. 70)	Non-Therapeutic state<br>Mean ± SD (n = 595 data points)	Therapeutic state<br>Mean ± SD (n = 756 data point)	*P*-value<br>(95% CI Difference)
**Demographics**			
Age (years)	54 (n=595)	54 (n=756)	1.000
Sex (Male/Female)	330/265	446/310	0.212
Height (cm)	164.29 ± 12.11	164.65 ± 13.58	0.613 (-1.85 to 1.13)
Weight (kg)	69.26 ± 12.92	69.40 ± 14.27	0.854 (-2.57 to 2.29)
**Hematology**			
White blood cell count ($\times 10^3$/uL)	9.60 ± 3.98	9.43 ± 3.64	0.423 (-0.24 to 0.58)
Segment neutrophil (%)	86.58 ± 7.75	84.64 ± 8.04	<0.001 (1.09 to 2.79)
Lymphocyte (%)	7.29 ± 5.46	8.46 ± 5.54	<0.001 (-1.76 to -0.58)
Monocyte (%)	5.18 ± 2.79	5.73 ± 2.83	<0.001 (-0.85 to -0.25)
**Renal Function**			
BUN (Blood urea nitrogen) (mg/dL)	35.48 ± 20.15	27.88 ± 16.89	<0.001 (5.58 to 9.62)
Creatinine (mg/dL)	3.12 ± 2.75	1.96 ± 1.98	<0.001 (0.90 to 1.42)
eGFR-MDRD -IDMS (mL/min/1.73 m2)	39.72 ± 30.66	55.91 ± 30.89	<0.001 (-19.50 to -12.88)
eGFR-CKD-EPI (mL/min/1.73 m2)	43.22 ± 32.79	58.33 ± 30.49	<0.001 (-18.53 to -11.69)
**Electrolytes and Metabolites**			
Uric acid (mg/dL)	4.83 ± 1.98	4.43 ± 1.99	<0.001 (0.19 to 0.61)
Potassium (mmol/L)	4.20 ± 0.64	4.40 ± 0.67	<0.001 (-0.27 to -0.13)

Chloride (mmol/L)	104.41 ± 5.58	105.71 ± 4.85	<0.001 (-1.87 to -0.73)
Phosphorus (mg/dL)	3.41 ± 1.89	2.72 ± 1.52	<0.001 (0.50 to 0.88)
Mg (mg/dL)	1.94 ± 0.35	1.83 ± 0.31	<0.001 (0.07 to 0.15)
Osmolality, serum (mOsm/kg)	296.84 ± 10.21	294.03 ± 9.42	<0.001 (1.75 to 3.87)

Data are presented as mean ± standard deviation (SD) for continuous variables and counts (percentages) for categorical variables. 95% confidence intervals (CI) for the difference between groups are provided where applicable. For comparisons between the Non-Therapeutic and Therapeutic Tacrolimus Concentration (6~10 ng/ml) Groups, normally distributed continuous variables were analyzed using an independent t-test. Categorical variables were compared using the Chi-square test.
